# Access to environmental health assets across wealth strata: Evidence from 41 low- and middle-income countries

**DOI:** 10.1371/journal.pone.0207339

**Published:** 2018-11-16

**Authors:** Jay P. Graham, Maneet Kaur, Marc A. Jeuland

**Affiliations:** 1 Public Health Institute, Oakland, California, United States of America; 2 Johns Hopkins Bloomberg School of Public Health, Baltimore, Maryland, United States of America; 3 Duke University, Durham, North Carolina, United States of America; 4 RWI-Leibniz Institute for Economic Research, Essen, Germany; The University of Warwick, UNITED KINGDOM

## Abstract

**Introduction:**

Low levels of household access to basic environmental health assets (EHAs)–including technologies such as clean cookstoves and bed nets or infrastructure such as piped water and electricity–in low- and middle-income countries (LMICs) are known to contribute significantly to the global burden of disease. This low access persists despite decades of promotion of many low-cost, life-saving technologies, and is particularly pronounced among poor households. This study aims to characterize variation in access to EHAs among LMIC households as a function of wealth, as defined by ownership of various assets.

**Methods:**

Demographic and Health Survey (DHS) data from 41 low- and middle-income countries were used to assess household-level access to the following EHAs: 1) improved water supply; 2) piped water supply; 3) improved sanitation; 4) modern cooking fuels; 5) electricity; and 6) bed nets. For comparison, we included access to mobile phones, which is considered a highly successful technology in terms of its penetration into poor households within LMICs. Ownership levels were compared across country-specific wealth quintiles constructed from household assets using bivariate analysis and multivariable linear regression models.

**Results:**

Access to EHAs was low among the households in the bottom three quintiles of wealth. Access to piped water, modern cooking fuels, electricity and improved sanitation, for example, were all below 50% for households in the bottom three wealth quintiles. Access to certain EHAs such as improved water supply and bed nets increased only slowly with concomitant increases in wealth, while gaps in access to other EHAs varied to a greater degree by wealth quintile. For example, disparities in access between the richest and poorest quintiles were greatest for electricity and improved sanitation. Rural households in all wealth quintiles had much lower levels of access to EHAs, except for bed nets, relative to urban households.

**Conclusions:**

The findings of this study provide a basis for understanding how EHAs are distributed among poor households in LMICs, elucidate where inequalities in access are particularly pronounced, and point to a need for strategies that better reach the poor, if the global environmental burden of disease is to be reduced.

## Introduction

It has been estimated that the environmental burden of disease–including exposure to contaminated water, poor sanitation and hygiene, household air pollution from cooking and heating with solid fuels, ambient air pollution, etc.–contributes to an estimated 23% of global deaths and 26% of deaths among children under five [1]. This disease burden primarily consists of respiratory diseases, which are responsible for an estimated 2 million premature deaths and diarrheal diseases, causing approximately 700,000 deaths and 2 billion cases annually [2]. In 2013, there were also an estimated 198 million malaria cases worldwide, leading to 584,000 deaths [3]. The burden of these diseases and other environmental health illnesses disproportionately affects low- and middle-income countries (LMICs) [1].

Environmental health assets (EHAs) that include piped water and sanitation facilities, and other technologies such as clean cookstoves, bed nets, and point-of-use water treatment devices, are recognized solutions that can enhance quality of life and reduce these health burdens [4, 5]. Many, but not all, of these effective technologies are considered to be cheap, and relatively affordable even for the global poor [6]. In the last two decades, substantial efforts have therefore been devoted to scaling-up access to EHAs. Examples of these efforts include the Millennium Development Goals and the more recent Sustainable Development Goals, which set clear targets for water, sanitation and hygiene (WASH). These include reducing the proportion of the global population without access to safe drinking water sources and improved sanitation as well as targets to reduce the incidence of malaria through increased use of bed nets [7]. Furthermore, in 2010, a global public-private partnership was established that set a target for 100 million households to adopt clean and efficient cookstoves and fuels by 2020 [8]. The Sustainable Development Goals (SDGs) meanwhile aim to build on the momentum of increased access to a range of live-saving environmental health infrastructure and technologies [9]. The adoption of SDG number 7.1, for example, aims to ensure access to affordable, reliable, and modern energy for all by 2030 [10]. This highlights a high level of political recognition for energy’s role in development. Between 2000 and 2016, an estimated 1.2 billion people gained access to electricity but approximately 1.1 billion people still remained without access to electricity at the end of that period. In terms of clean cooking, roughly 2.8 billion lack access to clean cooking options—2.5 billion people use solid biomass as their primary fuel for cooking, while 120 million use kerosene and 170 million use coal [10]. Population growth in LMICs, especially sub-Saharan Africa, has resulted in increases in use of solid biomass by about 400 million people since 2000 [10]. Affordability remains a major barrier, and capital costs for off-grid systems put cleaner options out of reach for the poor [10].

While it might appear obvious that household access to EHAs would vary with wealth, there is value in considering these patterns carefully. On the one hand, many policies and strategies to promote EHAs attempt to target the poor. Many of these technologies, and especially infrastructure solutions (e.g., electricity and piped water or sewer), are deployed with substantial government investment, and there is often an attempt to then create pro-poor tariffs for these services. Thus, one might anticipate that public funding would reduce disparities in access between wealthier versus poorer households [11, 12], although some have questioned whether so-called “lifeline” tariffs really reach the poor [13]. Other EHAs, such as bed nets, have typically been distributed for free such that access should be equitable [14, 15]. For those EHAs perceived as items that households are generally responsible for purchasing themselves (toilets or clean fuels, for example), greater disparities in access might be expected between wealth groups. These EHAs, however, are often subsidized which may diminish the importance of household wealth [16, 17].

On the other hand, many development programs attempt to apply market-based approaches to increase access to many EHAs, especially those that are considered purchasable by households (e.g. drinking water filters, toilets and clean cookstoves) [18–23]. The shift towards market oriented approaches stems from a desire to create financially viable models that meet consumers’ demands and can grow organically over time [24]. Implementers of these programs often highlight the challenges that governments, non-governmental organizations (NGOs), and international development programs have faced in delivering effective and sustainable dissemination programs [25, 26]. Market strategies instead focus on the idea that “entrance of the private sector …[will] improve the efficiency relating to business development and technology production, access, and diffusion” [21, 27]. Research suggests that market-based approaches can facilitate the engagement of new agents and corporate actors, leveraging financial and technical capacities in new ways. Still, it is unclear what these strategies imply for access across different wealth groups [28–30].

Some researchers have noted that market-based approaches may bypass the poor [31, 32], who arguably have the greatest need for EHAs. Proponents of market-based EHAs increasingly respond that “pro-poor” market-based approaches are possible, and often point to the nearly “ubiquitous cell phone coverage within the BoP [bottom of the pyramid] population” [33]. These anecdotes aside, information and knowledge gaps remain in our understanding of who has access to EHAs and how poverty relates to this access [32, 34]. Analyzing disparities in access to EHAs by wealth and other demographic variables is an important step towards developing an evidence-based understanding of the types of interventions and subsidies that may be required to expand access of EHAs among the poor. Such understanding could potentially be used to enhance strategies that address persistent gaps in access to EHAs among the poor.

Limited prior research has compared access to a variety of EHAs to understand how access is differentially affected by poverty levels [35]. This paper presents a multi-country analysis of the relationship between access to EHAs and household wealth. We cover more technologies than previous studies, and present evidence from a consistent set of surveys recently deployed across a wide range of low and middle-income countries. The analysis focuses on access to improved water, improved sanitation, electricity, and improved cooking fuels in LMICs. In presenting these results, this paper aims to provide new and comprehensive evidence on the question of differential access to EHAs among the poor. This analysis will likely be useful for informing a more nuanced understanding of persistent gaps in access.

## Methods

In order to explore the relationship between wealth and the ownership or presence of EHAs, this study utilized data from the Demographic and Health Survey (DHS) Program. Datasets were obtained for LMICs, as defined by the 2015 World Bank “Country and Lending Groups” [36]. Countries– 41 in all–were first screened for inclusion based on having a Standard DHS between 2008 and 2013, due to the greater availability and comparability of surveys within that time period (the original analysis was carried out in early 2015, and data from later than 2014 was not available at that time). For these 41 countries, we included additional DHS datasets if another existed after the year 2000–29 of the 41 original countries had a second dataset that we could use–to allow us to incorporate changes in asset ownership over time (and surveys from 2014–2017 were subsequently added to this original analysis, as they became available). Of the 12 original countries for which we did not add a second dataset, 6 did not have one at all, and 6 countries with a second dataset were excluded due to changes in regional identifiers that precluded construction of consistent analyses using the methods described further below. The full list of included countries (41) and surveys (70) is provided in supporting information (S1 Table).

The DHS survey uses a two-stage cluster sampling procedure. In the first step, the country is stratified into geographic regions. Then, using probability sampling proportional to size, clusters are selected for inclusion within each of those regions. These clusters, or primary sampling units (PSUs), are usually small villages in rural areas and neighborhoods in urban areas. Within a selected PSU, households are randomly sampled for surveying. In order to improve statistical inference and data collection logistics, the survey over-samples in regions with small populations and under-samples in regions with large populations. For countries with multiple rounds of DHS data, different households are sampled in each DHS wave; it is not a panel dataset.

The outcome variables of interest are households’ access to the following infrastructure or technological improvements: 1) improved water supply; 2) piped water supply; 3) improved sanitation; 4) improved fuels for cooking; 5) electricity; and 6) bed nets (Table 1). For comparison with a highly successful non-health technology, access to a mobile phone (i.e. anyone in the household with access) was also assessed. Improved water source included: household connection to piped water, access to a public standpipe, borehole, protected dug well, protected spring, or rainwater collection [37]. Improved sanitation included: connection to a public sewer, connection to a septic system, pour-flush latrine, simple pit latrine, and ventilated improved pit latrine [37]. For this analysis, improved fuels included only LPG given the evidence about the negative health effects of kerosene, though we note that results including kerosene were not substantively different [38]. The socio-demographic variables considered in the analysis of ownership of these EHAs included: other household asset measures, year of survey and survey phase, household’s urban/rural status, religion, ethnicity, language, household size, number of children, ownership of animals and head of household characteristics: education, marital status, sex, and age.

**Table 1:** Variables and descriptive statistics, overall and for urban and rural subsamples.

	29 countries with 2 rounds; Both rounds				All 41 countries; DHS from 2008–2013 only			
Variable	Overall	N	Urban	Rural	Overall	N	Urban	Rural
**Basic characteristics**								
Urban (%)	38	898,570			36	559,464		
Female head of HH^1^ (%)	23	898,567	25	22	24	559,462	26	23
Age of head of HH, mean ± SE	45.7 ± 15.5	897,791	44.8 ± 14.9	46.3 ± 15.8	45.6 ± 15.5	559,013	44.8 ± 14.9	46.1 ± 15.8
Primary education, head of HH (%)	53	896,303	55	51	70	555,725	82	63
Secondary education—head of HH (%)	30	896,303	45	21	36	555,725	56	25
Tertiary education—head of HH (%)	9.2	896,303	18	4.0	9.5	555,725	19	4.3
Married head of HH (%)	14	841,254	14	14	18	501,440	18	18
Never married head of HH (%)	4.6	841,254	7.0	3.1	5.5	501,440	8.9	3.7
HH Size, mean ± SE	4.87 ± 2.90	898,570	4.62 ± 2.88	5.03 ± 2.91	4.93 ± 2.91	559,464	4.67 ± 2.90	5.08 ± 2.91
HHs with children (%)	80	898,569	66	89	84	559,463	68	94
**Access to improved technologies**								
Electricity (%)	50	840,405	79	33	47	537,710	78	30
Piped water (%)	25	882,366	41	14	22	559,194	39	12
Improved water (%)	68	882,249	78	62	67	559,077	78	61
Improved sanitation (%)	39	878,913	52	30	36	559,261	51	28
Bed net (%)	53	549,247	52	54	62	353,673	61	63
Chimney hood (%)	15	67,062	16	14	11	95,240	11	11
Improved fuel^2^ (%)	17	845,635	35	5.8	18	540,316	36	7.3
Charcoal (%)	14	845,635	21	10	11	540,316	22	4.3
Mobile phone (%)	63	783,842	82	52	62	559,104	83	50
Treat water (%)	32	797,764	32	32	30	532,064	31	29
Hand washing place in HH (%)	51	491,589	61	45	54	350,647	61	49
**Wealth indices**								
Wealth quintile from DHS, mean	2.95 ± 1.43	898,570	2.34 ± 1.21	3.96 ± 1.17	2.95 (1.43)	559,464	4.00 (1.15)	2.35 (1.22)
Wealth quintile using all variables^3^, mean	2.92 ± 1.44	898,048	2.86 ± 1.42	3.02 ± 1.46	2.93 (1.44)	559,259	2.98 (1.48)	2.90 (1.41)

^1^HH refers to household.

^2^Improved fuel includes LPG, but not kerosene

^3^The wealth index used here is a country-specific index that was constructed using the first principle component obtained using PCA over all asset variables included in that country’s survey, only excluding the outcome variables.

These variables were checked for consistency, and when necessary, renamed, or recoded to appear in common units. Some specific variables that required recoding included land and livestock ownership, specific categories of household members, sources of drinking water, sanitation type, and some measures of assets. Few countries provided information on religion or ethnicity, thus, these variables were not included in the analysis.

### Construction of wealth indices

Next, because the assets provided in each country dataset are not identical across all countries in the DHS, several measures of household wealth were created that included varying subsets of the asset variables. The DHS includes a wealth index specific to each country that is constructed using principal components analysis (PCA), however, this index was not appropriate for our purposes because it includes the measures that are analyzed as outcomes, e.g., drinking water source and sanitation type, primary cooking fuel used, mobile phone ownership and bed net ownership [39].

In this analysis, we thus developed several alternate wealth indices: 1) country-specific wealth indices based on all possible asset variables for each country that were not analyzed as outcomes (this index is least comparable across countries but is based on the highest number of asset variables); 2) country-specific wealth indices based on the subset of such variables that were available in more than 75% of the original 41 country datasets; 3) country-specific wealth indices based only on the subset of such assets that were available across all countries; and 4) overall wealth indices constructed from the pooled country datasets based only on the subset of assets that were available across all countries. These final two indices are most comparable across countries but are based on the lowest number of asset variables. Assets with low variation (<2% or >98% ownership across countries) were excluded from construction of the indices. The wealth variable in each case was constructed by extracting the first principal component obtained using PCA. The percent variance explained ranged from approximately 10–50% depending on the various definitions of assets described above. As expected, the percent of the variance explained by this first principal component was higher for the index with the fewest assets, i.e., that containing only assets included in all country datasets (Table 2).

**Table 2:** Variation explained by the first principal component of each wealth index^1^.

	All 41 countries–DHS from 2008–2013^2^			29 countries– 2^nd^ DHS survey wave since 2000^3^		
VARIABLES	Country w/lowest variation explained	Country w/median variation explained	Country w/highest variation explained	Country w/lowest variation explained	Country w/median variation explained	Country w/highest variation explained
**Country-specific measures**						
Wealth quintile–all assets	0.11	0.20	0.32	0.12	0.20	0.32
	(Kyrgyz)	(Burkina Faso)	(Tanzania)	(Armenia)	(Bangladesh)	(Tanzania)
Wealth quintile–only assets	0.13	0.22	0.32	0.14	0.22	0.32
included for 75% of countries	(Kyrgyz)	(Nepal)	(Tanzania)	(Armenia)	(Madagascar)	(Tanzania)
Wealth quintile–only assets	0.23	0.34	0.46	0.26	0.34	0.46
included for all countries	(Tajikistan)	(Nepal)	(Ethiopia)	(Egypt)	(Nepal)	(Lesotho)
**Global measure**						
Wealth quintile–only assets	0.29			0.35		
included for all countries						

^1^ The wealth indices were constructed using the first principal component obtained using PCA over different subsets of asset variables, only excluding the outcome variables, as described in the text.

^2^ Percent of variation explained by the first component for only DHS conducted between 2008 and 2013.

^3^ Percent of variation explained by the first component for the second DHS survey conducted after 2000.

In addition, because this analysis is concerned with how relative wealth relates to differences in access to public health infrastructure for both urban and rural households, for whom asset ownership can be quite different due to the influence of livestock and land ownership, the PCA was performed separately for three different samples of households from the DHS: 1) urban, 2) rural, and 3) the pooled sample. Then, in order to create a wealth index that allowed for more appropriate comparison of relative wealth across both urban and rural quintiles (rather than overall national wealth quintiles) in the pooled sample, the urban score obtained from subsample 1 was regressed on the pooled sample score, and the same was done for the rural score for subsample 2 [39]. The coefficients from these models were then used to create a pooled score, as specified below:

$$W_{in}^{pooled}={\beta_{0}}^{u}+{\beta_{1}}^{u}∙W_{in}^{u},$$ (1)

$$W_{in}^{pooled}={\beta_{0}}^{r}+{\beta_{1}}^{r}∙W_{in}^{r},$$ (2)

where $W_{in}^{pooled}$ is the combined sample wealth index for household *i* in country *n*, $W_{in}^{s}$ is the wealth index for household *i* if it is in stratum *s* (rural *r* or urban *u*), and *β*_*j*_^*s*^ are coefficients obtained from the regression described above. This approach is consistent with that used by the DHS for construction of its wealth indices [39].

Finally, the wealth indices for each country were converted into quintiles and the sampling weight was applied in order to make the quintiles representative of the actual household wealth distribution within each country for the given survey year.

### Statistical analyses

We first employed bivariate analyses to analyze the associations between each infrastructure/technology and wealth quintile, using Kruskal Wallis chi-square tests. We then conducted multivariable linear regression with year and country fixed effects to adjust for the differences across countries and over time. The basic estimating equation used to estimate the relationship between the access variables and wealth for our preferred specification was:

$$Y_{irnt}=\beta_{0}+\beta_{1}∙W_{irnt}+\beta_{2}∙U_{irnt}+\beta_{3}∙X_{irnt}+\tau_{t}+\eta_{r}+\varepsilon_{in},$$ (3)

where *Y*_*irnt*_ is a binary indicator that is equal to 1 if a household *i* living in region *r* of country *n* in year *t* has access to an infrastructural or technological improvement (see outcomes listed above), and 0 otherwise. *W*_*irnt*_ is the wealth quintile, *U*_*irnt*_ is an indicator that is equal to 1 for urban households, and *X*_*irnt*_ is a vector of other control variables that include head of household characteristics (gender, age, education and marital status), and household demographics (size of the household and number of children). *τ*_*t*_ and *η*_*r*_ are year and region fixed effects, and *ε*_*irnt*_ is a normally-distributed error term. All standard errors are clustered at the country level. Our base specification only includes the set of 29 countries with multiple rounds of DHS data after 2000. It also includes the country-specific wealth indices based on all possible asset variables for each country, because we are especially interested in whether the associations between coverage and wealth hold within countries. We comment on the sensitivity of the results to other definitions of wealth in the text, and also on estimation that uses only the original round of data from the 41 countries with a DHS conducted between 2008 and 2013. Further, we used the Oaxaca-Blinder decomposition to more carefully analyze the extent to which differences in EHA ownership were driven by disparities in wealth, versus other determinants that are correlated with wealth. Then, to better assess urban and rural differences, we conducted a separate analysis in which we replaced the two *W*_*irnt*_ and *U*_*irnt*_ terms with a vector of nine indicator variables for each wealth quintile in rural and urban areas (where the rural poor serve as the reference category).

In addition, we estimated a random intercept and slope model (with independent variance parameters), allowing both the base level of access (intercept) and the association with wealth (slope) to vary by country, controlling for the other covariates described above. This analysis, which includes year but not region fixed effects, provides greater insight on the degree to which the relationship between wealth and access varies in strength across countries in the overall sample.

Finally, we constructed a smaller dataset that transformed that with repeated cross sections of households to one that is a panel of regions. In the specification of that model, we estimated a modified equation as shown below:

$$Y_{rnt}=\beta_{0}+\beta_{1}∙W_{rnt}+\beta_{2}∙U_{rnt}+\beta_{3}∙X_{rnt}+\tau_{t}+\eta_{r}+\varepsilon_{in}.$$ (4)

The main advantage of this analysis is to better control for unobserved differences that may confound the household-level results. The regional fixed effects absorb all unobserved time-invariant differences that explain EHA ownership. This analysis comes with a cost of greatly reduced power, however, and provides estimates on how changes in average regional wealth (and other independent variable) levels are correlated with changes in average regional EHA ownership, rather than the household-level analog. Since variation in the former is much lower than variation in the latter, using the regional variables also reduces power.

### Hypotheses

At the inception of the analysis, we hypothesized that:

1. EHA ownership would be positively related to wealth;
2. EHA ownership would be higher among urban households than among rural ones, especially for capital-intensive infrastructure such as piped water and electricity; and
3. Within the urban or rural subsamples, EHAs that are often provided with substantial government or donor support (e.g., piped water, electricity, improved water and sanitation, and bed nets) would be more equitably distributed across wealth quintiles than those that receive less such support (e.g., improved fuels and mobile phones).

Our analyses were structured to shed light on each of these hypotheses.

## Results

### Access to EHAs by wealth

**Fig 1:**
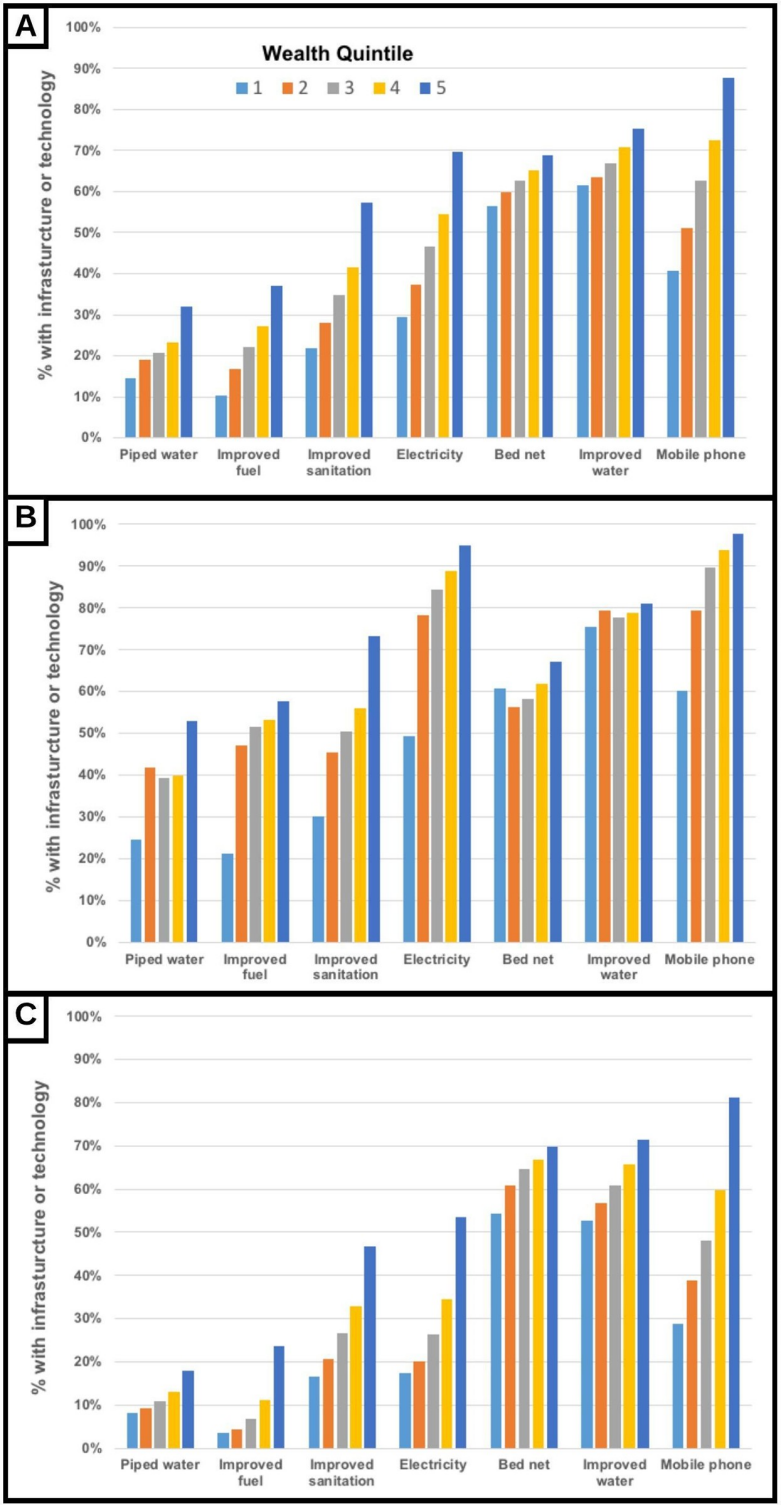
A) Relationship between wealth quintile and coverage with different environmental health assets for all households; B) Relationship between wealth quintile and coverage with different environmental health assets for urban households; C) Relationship between wealth quintile and coverage with different environmental health assets for rural households.

There were major differences in access to the EHAs included in the study. For every EHA variable considered in this analysis, household access is positively associated with wealth in the bivariate analyses (Fig 1 Panel A). The results that are shown use the country-specific wealth indices that we constructed based on all possible household asset variables for each of the 29 countries with multiple datasets (the graphs for the 41 countries with data from 2008–2013 are qualitatively similar). Though all associations are highly significant (p<0.01 in the Kruskal Wallis Chi-square tests), the rate of increase across wealth groups varies. For example, bed net ownership and access to improved water sources increase only slowly with wealth, rising by about 14 percentage points over the five wealth quintiles. In comparison, electricity, and improved sanitation rise more steeply with wealth, increasing by 42 and 38 percentage points, respectively. Similarly, mobile phones increased 46 percentage points over the five wealth quintiles. One interesting observation that emerges from these differences in the wealth gradient of access is that mobile phone coverage is lower than bed net ownership and access to improved water for the bottom two quintiles, but higher than the latter two in the top two quintiles. Additionally, access to many of these EHAs is very low among the poorest households. Access to piped water, improved fuel, and improved sanitation for example are all below 40% for households in the bottom three wealth quintiles, and below 50% among these households for electricity. Findings were similar for the other wealth indices.

There is also a stark urban-rural divide in coverage rates (Fig 1 Panels B and C). Access among rural households to all EHAs, except bed nets, is lower across all wealth quintiles (p<0.01), and especially for the four with the lowest coverage rates overall: piped water, improved fuel, improved sanitation, and electricity. Among urban households, only the bottom wealth quintile has access below 40% for any technology (specifically piped water, improved fuel and improved sanitation), whereas coverage rates for these three improvements are below 40% in all but the highest wealth quintile in rural areas. In both urban and rural samples, coverage with improved water and bed nets are least correlated with increasing wealth. All wealth-infrastructure associations remain significant (p<0.01). In urban areas, only the poorest households (i.e. bottom quintile of wealth) have access to mobile phones that is below access to improved water. In rural areas, however, mobile phone access is well below coverage with these other technologies for all but the highest two quintiles.

### Multivariable analysis

We next estimate the EHA-specific associations with wealth, controlling for head of household characteristics and household demographics. We first estimate average slopes across all countries, and then, using a random slope and intercept model, allow for country-specific relationships in the intercept and wealth gradient of access. The average results, with region and year of survey fixed effects included, are summarized in Table 3. Coverage with all EHAs is positively associated with wealth. Consistent with the bivariate analyses, mobile phone, electricity, and improved sanitation coverage rates are most highly associated with wealth. Urban households have higher levels of coverage with all EHAs except for bed nets, and have especially higher access to electricity. The education variables are generally positively related to coverage as well, though notable differences exist between education attainment categories. Having a “previously married” status is associated with varying levels of access to EHAs. An increased number of children in the household is associated with less access to EHAs, except for bed nets and use of improved fuel. The wealth-related results are consistent across definitions of the wealth index (as summarized in Table 4) and sample countries (S2 Table), although the coefficient estimates for wealth are larger when using the global wealth index (rather than country-specific wealth). This is not surprising since poorer countries with lower EHA coverage have lower average wealth when using the global index.

**Table 3:** Pooled multivariable regression estimates of the association between wealth and EHA access.

VARIABLES	Piped water	Improved water	Improved sanitation	Improved fuel	Electricity	Bed net	Mobile phone
Wealth quintile	0.035***	0.033***	0.076***	0.044***	0.085***	0.035***	0.091***
	(0.0043)	(0.0050)	(0.0092)	(0.0076)	(0.0099)	(0.0065)	(0.0080)
Urban	0.18***	0.17***	0.16***	0.16***	0.32***	0.017	0.20***
	(0.023)	(0.026)	(0.026)	(0.032)	(0.036)	(0.024)	(0.020)
Female head of HH	0.010**	0.019***	0.019***	0.0099**	0.018***	0.0088	-0.015**
	(0.0046)	(0.0050)	(0.0036)	(0.0037)	(0.0054)	(0.0051)	(0.0061)
Age of head of HH	0.0003**	0.0003	0.0022***	-0.0004**	-0.0003	-0.0009***	-0.0030***
	(0.0001)	(0.0002)	(0.0003)	(0.0002)	(0.0002)	(0.0003)	(0.0002)
Primary education–head of HH	0.0030	0.016**	0.012	-0.020**	0.012	0.035**	0.059***
	(0.0081)	(0.0078)	(0.0075)	(0.0079)	(0.011)	(0.014)	(0.012)
Secondary education–head of HH	0.031***	0.019**	0.063***	0.039***	0.052***	0.035***	0.088***
	(0.0067)	(0.0088)	(0.011)	(0.0097)	(0.010)	(0.0089)	(0.012)
Tertiary education–head of HH	0.058***	-0.030	0.12***	0.15***	0.059**	0.025*	0.066***
	(0.017)	(0.018)	(0.014)	(0.023)	(0.022)	(0.013)	(0.021)
Previously married–head of HH	0.0018	0.014***	-0.0079*	-0.0068	0.011*	-0.053***	-0.028***
	(0.0050)	(0.0038)	(0.0040)	(0.0052)	(0.0058)	(0.0071)	(0.0069)
Never married–head of HH	0.0074	-0.0081	-0.041***	-0.029	0.035***	-0.15***	-0.0003
	(0.0102)	(0.0153)	(0.012)	(0.018)	(0.0086)	(0.0098)	(0.0094)
Household size	-0.0005	0.0009	0.0096***	-0.007***	-0.0007	0.0020	0.020***
	(0.0012)	(0.0009)	(0.0013)	(0.0011)	(0.0014)	(0.0012)	(0.0013)
Number of children	-0.0076***	-0.008***	-0.015***	0.0014	-0.0088***	0.032***	-0.027***
	(0.0014)	(0.0014)	(0.0022)	(0.0013)	(0.0023)	(0.0047)	(0.0029)
Constant	0.035***	0.033***	0.076***	0.044***	0.085***	0.035***	0.091***
	(0.0043)	(0.0050)	(0.0092)	(0.0076)	(0.0099)	(0.0065)	(0.0080)
Observations	822,048	822,061	818,620	785,362	801,566	545,653	723,850
R-squared	0.473	0.217	0.366	0.477	0.616	0.258	0.420

Includes the 29 countries having 2 DHS surveys since the year 2000 (for analogous results from the 41 countries with a single round of data between 2008–2013, refer to S2 Table). The regression specification in this table is a linear regression that includes sub-national and year fixed effects, as described by Eq 2. Standard errors clustered at the country level are shown in parentheses. The wealth index used here is a country-specific index that was constructed using the first principle component obtained using PCA over all asset variables included in that country’s survey, only excluding the outcome variables. Significance of the coefficients is indicated as follows

*** p<0.01

** p<0.05

* p<0.1.

**Table 4:** Pooled multivariable regression estimates of the association between wealth and EHA access, across different definitions of the wealth index; for countries with multiple rounds (Panel A) and latest data from all countries (Panel B).

VARIABLES	Piped water	Improved water	Improved sanitation	Improved fuel	Electricity	Bed net	Mobile phone
PANEL A: 29 countries with more than 1 round since 2000							
Country-specific measures							
Wealth quintile—all assets from	0.035***	0.033***	0.076***	0.044***	0.085***	0.035***	0.091***
latest survey round	(0.0043)	(0.0050)	(0.0092)	(0.0076)	(0.0099)	(0.0065)	(0.0080)
Wealth quintile—all assets from	0.034***	0.034***	0.073***	0.042***	0.084***	0.033***	0.088***
original survey round	(0.0043)	(0.0050)	(0.0087)	(0.0074)	(0.0097)	(0.0059)	(0.0079)
Global measure							
Wealth quintile–only assets	0.041***	0.041***	0.070***	0.040***	0.090***	0.031***	0.091***
included for all countries	(0.0051)	(0.0047)	(0.0073)	(0.0087)	(0.0063)	(0.0045)	(0.0060)
PANEL B: All 41 countries with DHS between 2008–2013							
Country-specific measures							
Wealth quintile—all assets	0.036***	0.037**	0.066***	0.049***	0.086***	0.023***	0.10***
	(0.0042)	(0.0040)	(0.0074)	(0.0086)	(0.0085)	(0.0060)	(0.0049)
Wealth quintile–only assets	0.032***	0.036**	0.061***	0.043***	0.080***	0.019***	0.090***
included for 75% of countries	(0.0039)	(0.0041)	(0.0074)	(0.0085)	(0.0073)	(0.0054)	(0.0050)
Wealth quintile–only assets	0.033***	0.037**	0.059***	0.040***	0.082***	0.024***	0.092***
included for all countries	(0.0038)	(0.0030)	(0.0069)	(0.0076)	(0.0060)	(0.0050)	(0.0042)
Global measure							
Wealth quintile–only assets	0.041***	0.060***	0.062***	0.038***	0.11***	0.015***	0.10***
included for all countries	(0.0062)	(0.0047)	(0.0065)	(0.0069)	(0.0082)	(0.0045)	(0.0048)

Standard errors clustered at the country level are shown in parentheses, models include year of survey and region fixed effects (Panel A) or year of survey and country fixed effects (Panel B). The wealth indices were constructed using the first principal component obtained using PCA over different subsets of asset variables, only excluding the outcome variables, as described in the text. Significance of the coefficients is indicated as follows

*** p<0.01

** p<0.05

* p<0.1.

Results from a Oaxaca-Blinder decomposition that groups households into poor (bottom 2 quintiles) and non-poor (top 3 quintiles) demonstrates that differences in wealth endowments explain disparities in EHA ownership most strongly for piped water and improved fuel, followed by improved sanitation and electricity, relative to other differences across these two types of households (S3 Table). Across all assets, these other differences explain more of the gap in EHA ownership than wealth alone. Only for bed nets do the wealth endowments explain none of the difference in ownership.

The overall estimates of the association between wealth and EHA access remain stable even when allowing for country-specific intercepts and slopes (Table 5). In addition, the variances in the estimates of both of these parameters across countries are highly significant for all EHAs, which indicates that countries are heterogeneous in the degree to which disparities in coverage are related to disparities in wealth within countries. This may be due to differences in wealth inequality (more or less similar across wealth quintiles), may stem from differences in the relative costs of access across settings, or may relate to the differences in policies deployed to promote access in different countries. Cost differences could arise, for example, from differences in remoteness, population density, availability of labor, or other similar factors. Similar results apply in the sample of 41 countries with data from 2008–2013 (S4 Table).

**Table 5:** Pooled multivariable regression estimates of the association between wealth and EHA access, allowing for country-specific differences.

VARIABLES	Piped water	Improved water	Improved sanitation	Improved fuel	Electricity	Bed net	Mobile phone
Wealth quintile	0.039***	0.036***	0.072***	0.051***	0.092***	0.027***	0.095***
	(0.0056)	(0.0056)	(0.0070)	(0.0087)	(0.0089)	(0.0068)	(0.0066)
Urban	0.19***	0.17***	0.15***	0.18***	0.34***	-0.0041	0.22***
	(0.0274)	(0.029)	(0.026)	(0.032)	(0.042)	(0.030)	(0.022)
Constant	0.064	0.33***	-0.25***	-0.026	-0.011	0.28***	-0.087*
	(0.054)	(0.063)	(0.064)	(0.040)	(0.068)	(0.036)	(0.048)
Cross-country variance–wealth	0.0011***	0.0009***	00016***	0.0023***	0.0022***	0.0010***	0.0011***
	(0.00035)	(0.0002)	(0.0002)	(0.0004)	(0.0006)	(0.0004)	(0.0003)
Cross-country variance–constant	0.065***	0.048***	0.042**	0.038	0.095***	0.030***	0.032***
	(0.0.026)	(0.011)	(0.20)	(0.025)	(0.030)	(0.0083)	(0.008)
Observations	822,048	822,061	818,620	785,362	801,566	545,653	723,850

Includes the 29 countries having 2 DHS surveys since the year 2000 (for analogous results from the 41 countries with a single round of data between 2008–2013, refer to S4 Table). Standard errors clustered at the country level are shown in parentheses, models include head of household characteristics and household demographic controls, as well as year of survey fixed effects and random intercept and wealth slopes. The wealth index used here is a country-specific index that was constructed using the first principle component obtained using PCA over all asset variables included in that country’s survey, only excluding the outcome variables. Significance of the coefficients is indicated as follows

*** p<0.01

** p<0.05

* p<0.1.

To better isolate differences in wealth within urban and rural areas, the final analyses consider interactions between urban/rural residence and each level of wealth (Table 6). This analysis confirms the trends observed in the bivariate analysis. In particular, the gap across wealth quintiles is greatest in rural areas for mobile phones, electricity, and sanitation, and is especially steep between the highest two quintiles, suggesting that high levels of wealth are required to achieve the higher levels of ownership of these technologies. The gradient in rural areas for piped water and improved fuels is lowest, but this is largely because use of these technologies is so low. In urban areas, in contrast, the gradients are steepest for electricity, mobile phones, and sanitation, but the poorest quintile is the most disadvantaged relative to others, except in terms of access to improved water and bed nets.

**Table 6:** Pooled multivariable regression estimates of the association between wealth-urbanization interactions and EHA access.

VARIABLES	Piped water	Improved water	Improved sanitation	Improved fuel	Electricity	Bed net	Mobile phone
Wealth quintile 2*Rural	0.029***	0.049***	0.071***	0.0092	0.064***	0.061***	0.10***
	(0.0068)	(0.0083)	(0.017)	(0.0055)	(0.018)	(0.014)	(0.012)
Wealth quintile 3*Rural	0.040***	0.087***	0.13***	0.022**	0.13***	0.11***	0.19***
	(0.011)	(0.012)	(0.025)	(0.0091)	(0.029)	(0.021)	(0.020)
Wealth quintile 4*Rural	0.059***	0.13***	0.19***	0.039***	0.19***	0.13***	0.28***
	(0.014)	(0.016)	(0.033)	(0.012)	(0.041)	(0.024)	(0.025)
Wealth quintile 5*Rural	0.10***	0.20***	0.31***	0.11***	0.34***	0.17***	0.43***
	(0.017)	(0.023)	(0.046)	(0.027)	(0.051)	(0.030)	(0.034)
Wealth quintile 1*Urban	0.12***	0.22***	0.14***	0.052**	0.26***	0.068**	0.24***
	(0.018)	(0.024)	(0.028)	(0.022)	(0.039)	(0.026)	(0.020)
Wealth quintile 2*Urban	0.21***	0.26***	0.23***	0.13***	0.42***	0.083*	0.36***
	(0.026)	(0.028)	(0.053)	(0.041)	(0.043)	(0.041)	(0.019)
Wealth quintile 3*Urban	0.24***	0.27***	0.29***	0.20***	0.50***	0.098**	0.44***
	(0.032)	(0.031)	(0.061)	(0.050)	(0.057)	(0.043)	(0.029)
Wealth quintile 4*Urban	0.27***	0.27***	0.35***	0.27***	0.56***	0.12**	0.48***
	(0.033)	(0.032)	(0.059)	(0.057)	(0.057)	(0.049)	(0.033)
Wealth quintile 5*Urban	0.36***	0.26***	0.49***	0.38***	0.64***	0.16***	0.51***
	(0.043)	(0.037)	(0.046)	(0.051)	(0.049)	(0.043)	(0.035)
Constant	0.12***	0.33***	-0.15**	0.078***	0.14***	0.23***	0.15**
	(0.021)	(0.040)	(0.063)	(0.022)	(0.045)	(0.052)	(0.062)
Observations	822,048	822,061	818,620	785,362	801,566	545,653	723,850
R-squared	0.477	0.221	0.368	0.489	0.618	0.259	0.424

Includes the 29 countries having 2 DHS surveys since the year 2000. Standard errors clustered at the country level are shown in parentheses, models include head of household characteristics and household demographic controls, as well as year of survey fixed effects and region fixed effects. Wealth quintile 1*rural is the omitted category. The wealth index used here is a country-specific index that was constructed using the first principle component obtained using PCA over all asset variables included in that country’s survey, only excluding the outcome variables. Significance of the coefficients is indicated as follows

*** p<0.01

** p<0.05

* p<0.1.

We close this section commenting on the final analyses that used a regional panel for the 29 countries with multiple rounds of DHS data, rather than analyzing the repeated cross-sections of household data. While this analysis allows us to more carefully control for unobserved time-invariant differences that may confound the estimates of the relationship between wealth and EHA ownership, the sample size drops dramatically to between 382 to 579 regions, depending on the EHA being analyzed. It also offers a somewhat different interpretation as noted in the discussion below Eq 4, in that the variation of interest is in average changes in wealth within regions (which does not vary greatly), rather than household-level wealth. In this analysis, estimated coefficients remain positive and statistically different from zero for all EHAs except piped water and bed nets in a random effects specification that allows for cross-country differences in slopes and intercepts. The results are also broadly consistent with those obtained from the household-level random effects specification (Table 5). In a specification with regional fixed effects, the coefficient estimates remain positive, but are much smaller in magnitude; in addition, the fixed effects estimates are not statistically distinguishable from zero for any EHA. Urban EHA levels remain higher for most EHAs in the random and fixed effects models as well, though precision is again lost in the fixed effects specification (S5 Table). All in all, these regional results confirm that caution is warranted in assigning a causal interpretation to the associations that we document between wealth, urbanization and EHA access.

## Discussion

Though the environmental health community is well aware of disparities in susceptibility to environmental health illnesses and in access to EHAs that protect against them, there has been relatively little previous systematic presentation and comparison of the nature of the relationship between poverty and lack of access to different EHAs. This paper adds to this limited prior research by including a large range of environmental health assets and utilizing a wealth index rather than income and expenditure variables, with measures constructed from a consistent set of surveys. Our analysis also controlled for several potentially confounding variables, such as education, household size and number of children, to better characterize the relationship of wealth and access to EHAs. We found that access to EHAs is highly correlated with wealth: a higher percentage of wealthy than poor households had improved water, bed nets, electricity, in-house piped water connections, improved sanitation, and mobile phones. Our analysis also showed that access to EHAs is considerably higher in urban areas than in rural ones, except for bed nets. In addition, in urban areas, households in the poorest quintile were especially disadvantaged relative to others, while in rural areas, only the wealthiest quintile had relatively high access to EHAs.

Compared with prior related research [35], our study considered a broader set of countries and a greater range of EHAs with access rates that are measured using consistent surveys. We found in particular that access to piped water, improved fuels, and improved sanitation are all below 40% for households in the three poorest quintiles, and below 50% among these households for electricity. An interesting observation is that mobile phone coverage is lower than bed net ownership and access to improved water for the two poorest quintiles, but that the former is higher than the latter two in the two wealthiest quintiles. This finding challenges the often-heard idea that mobile phones have succeeded better than other technologies in reaching the poor in LMICs, because of the clear private benefits they provide.

Prior to analyzing the data, we expected less pronounced disparities in access to environmental health infrastructure that is often developed with the help of government investment, such as electricity and piped water. We also predicted that bed net access would be fairly equitable given distribution models that often provide these for free. However, these infrastructure and distribution hypotheses (equitable access to electricity and piped water) were not supported by the data. Household wealth is indeed highly correlated with access to all EHAs, though the relationship is somewhat weaker in the case of bed nets. The specific reasons behind these disparities in access are surely complex, and likely span issues such as errors in the targeting of subsidies, higher costs in reaching particularly low income households and high connection fees, poor households’ inability to avail of subsidies due to lack of awareness, as well as other issues. Given the differences across urban and rural areas for households with similar levels of wealth, it seems likely that targeting EHA delivery in rural areas will face particular challenges. Price is not the only impediment in rural areas, since many relatively well-off households still do not own and use these technologies.

Thus, while the empirical findings in the literature point to a range of social and individual factors that influence whether households in LMICs will adopt new EHAs, the financial constraints that households face should receive equal or greater attention. Prior comparative studies have shown that income is a significant predictor of rates of access to and adoption of products and services [35, 40]. Additionally, microeconomic technology-specific studies have given us important insights into the role of income, price, and access to credit in the adoption of technologies. Households in the highest socioeconomic status quintiles show greater rates of adoption of products [41, 42]. Furthermore, as the size of a subsidy increases and/or the price of a product decreases, the rate of adoption has been shown to increase among low income households [43–46]. There is evidence that access to credit and liquidity constraints limit the adoption of new products [47, 48]. Finally, consistency of income across time has also been shown to positively influence the likelihood of access to improved sanitation, which is likely linked to confidence in recuperating the expenditures made on sanitation [49].

There were limitations to this study. First, the ability of the assets used in this analysis to measure poverty were restricted in their explanatory power. In general, the capacity of a set of assets to differentiate levels of poverty varies significantly across LMICs and over time within a country because of differences in access to a variety of assets. It would be useful to apply different indices to different countries and within a country over time, but this was not desirable for our study that aimed to compare inequalities across countries. Second, due to data limitations, our analysis focused on ownership of assets rather than use of these technologies. There are many reasons why ownership of environmental health assets might not generate health improvements, but incomplete use (e.g., stacking of stoves and fuels, collection of water from improved and unimproved sources, partial use of bed nets) is certainly important among them. Thus, these ownership variables should not be considered to fully explain many environmental health disparities. Third, the asset-based approach to measuring wealth is a proxy measure of relative wealth rather than absolute wealth, which can only be used to assess a household’s ranking within a gradient across a population.

Many researchers have found that household poverty does not provide a complete story of adoption, however. Intra-household power dynamics can affect access, and researchers have found that women's decision making power for major household purchases is positively associated with households having better sanitation [50]. These and other similar findings suggest that increased gender equity could potentially have spillover effects that result in more households opting to improve access to EHAs. Education levels, especially of the primary decision maker in the household, may be critical for processing information and determining if the benefits of a particular technology outweigh the costs. Low levels of education are generally found to decrease understanding of the advantages and disadvantages of new products and are associated with less awareness of the possibilities of innovation [48]. The information that a household is able to access related to the technology can also be important. Thus, empirical studies find that adoption increases when paired with promotional activities that highlight the benefits of the product and that imperfect information limits the adoption of products [41, 47, 51]. In fact, many studies point to education and socio-economic status as the two biggest influences on access to EHAs related to sanitation, water, and clean cooking, within and across countries [49, 52–56].

Previous studies have also documented a stark urban-rural divide in access to environmental health improvements [57], that is also evident in the cross-country dataset discussed in this paper. It is likely that costs are higher in remote rural areas; previous monitoring results in the area of water and sanitation have found such issues to be important [52, 58]. In our analysis, urban residence was positively and significantly associated with access to all of the technologies, with the exception of bed nets. It may be that malaria programs that provide bed nets have been more effective at reaching rural populations in contrast to programs aiming to increase access to other technologies. The results highlight, however, the difficulty in reaching more remote populations with environmental health technologies.

Overcoming these–financial, educational, and remoteness–constraints will likely require more than simple demand stimulation and market provision of these EHAs. End-user subsidies, supply-chain support, information campaigns, and educational investments thus seem essential, if disparities in access to EHAs are to be reduced. Still, researchers and proponents of greater use of market approaches have noted that having the right product available strongly influences consumers’ willingness to purchase EHAs [31, 59]. Dumpert and Perez, for example, state that “consumers are willing to pay for high-quality aspirational products (e.g., toilets) even if lower cost options are available.” The authors go on to say: “In these circumstances, the bottlenecks had less to do with stimulating demand and more to do with the absence of a technology, service, and/or means of financing that helped consumers achieve their product aspirations.” Meanwhile, public- or donor-driven programs often face important demand-side challenges as well, when conditions in target beneficiary communities are not conducive to adoption and continued use of new technologies [6]. Thus, it seems clear that market solutions can play an important role in distributing EHAs that better meet households’ needs. These advantages notwithstanding, such solutions will likely face challenges in reaching the poor.

## Conclusion

This paper provides a unique analysis of inequalities in access to environmental health assets. While it is generally understood that wealthier individuals have greater access to public health goods and services than their poorer counterparts, this paper shows that the relationships between poverty and access to such EHAs are robust and strong. Even so, access is more equitably distributed for some types of EHAs–notably improved water and bed nets–than others. The extremely low access to EHAs among the poorest households in our study highlights the need for continued efforts to target the poor. Recently, the inequality of access to EHAs has gained more attention as decision-makers have come to regard averages within a country as an inadequate summary of a country’s performance in meeting global targets. More effort is needed to understand why inequity varies across different EHAs, in order to identify effective levers for improving access to critical public health technologies and infrastructure among the poor.

## Supporting information

S1 Table List of countries included in the analysis. (DOCX)

S2 Table Pooled multivariable regression estimates of the association between wealth and EHA access. (DOCX)

S3 Table Oaxaca-Blinder decomposition of the role of wealth disparities in differences of EHA ownership. (DOCX)

S4 Table Pooled multivariable regression estimates of the association between wealth and EHA access, allowing for country-specific differences. (DOCX)

S5 Table Pooled multivariable regression estimates of the association between wealth and EHA access, from regional-level panel analysis. (DOCX)
