# Supplementary material for: Access to environmental health assets across wealth strata: Evidence from 41 low- and middle-income countries
Source: PLoS One. 2018 Nov 16;13(11):e0207339. doi: 10.1371/journal.pone.0207339 (PMC6239312; doi:10.1371/journal.pone.0207339)
Supplement: S1 Table — (DOCX) [file pone.0207339.s001.docx]

**S1 Table.** List of countries included in the analysis

| Country | Year of DHS used for 41 country analysis | Year of most recent DHS | Year of prior DHS | Missing EHAs in prior DHS | Included in 29 country analysis? | Reason for exclusion from 29 country analysis | Other notes |
| --- | --- | --- | --- | --- | --- | --- | --- |
| Armenia | 2010 | 2015-16 | 2010 | n.a. | Yes | n.a. |  |
| Bangladesh | 2011 | 2014 | 2011 | n.a. | Yes | n.a. |  |
| Democratic Republic Congo | 2007 | 2013-14 | 2007 | n.a. | Yes | n.a. |  |
| Egypt, Arab Rep. | 2008 | 2014 | 2008 | n.a. | Yes | n.a. |  |
| Ethiopia | 2011 | 2016 | 2011 | n.a. | Yes | n.a. |  |
| Ghana | 2008 | 2014 | 2008 | n.a. | Yes | n.a. |  |
| Honduras | 2011-12 | 2011-12 | 2005-06 | n.a. | Yes | n.a. |  |
| Haiti | 2012 | 2012 | 2005-06 | n.a. | Yes | n.a. |  |
| Kenya | 2008-09 | 2014 | 2008-09 | n.a. | Yes | n.a. |  |
| Liberia | 2013 | 2013 | 2007 | n.a. | Yes | n.a. | 1 inconsistent region dropped |
| Lesotho | 2009 | 2014 | 2009 | n.a. | Yes | n.a. |  |
| Malawi | 2010 | 2015-16 | 2010 | n.a. | Yes | n.a. |  |
| Nigeria | 2013 | 2013 | 2008 | n.a. | Yes | n.a. |  |
| Nepal | 2011 | 2016 | 2011 | n.a. | Yes | n.a. |  |
| Philippines | 2013 | 2013 | 2008 | n.a. | Yes | n.a. |  |
| Rwanda | 2010 | 2014-15 | 2010 | n.a. | Yes | n.a. |  |
| Sierra Leone | 2013 | 2013 | 2008 | n.a. | Yes | n.a. |  |
| Timor-Leste | 2009-10 | 2016 | 2009-10 | n.a. | Yes | n.a. |  |
| Tanzania | 2010 | 2015-16 | 2010 | n.a. | Yes | n.a. | 4 inconsistent regions dropped |
| Benin | 2011-12 | 2011-12 | 2006 | Electricity | Yes | n.a. |  |
| Bolivia | 2008 | 2008 | 2003 | Sanitation; mobile phone | Yes | n.a. |  |
| Indonesia | 2012 | 2012 | 2007 | Mobile phone | Yes | n.a. |  |
| Cambodia | 2010 | 2014 | 2010 | Water; mobile phone | Yes | n.a. |  |
| Mozambique | 2011 | 2011 | 2003 | Mobile phone | Yes | n.a. |  |
| Pakistan | 2012-13 | 2012-13 | 2006-07 | Mobile phone | Yes | n.a. | 2 inconsistent regions dropped |
| Burkina Faso | 2010 | 2010 | 2003 | Mobile phone | Yes | n.a. | Missing marital status; 1 inconsistent region dropped |
| Cameroon | 2011 | 2011 | 2004 | Mobile phone | Yes | n.a. | Missing marital status |
| Guinea | 2012 | 2012 | 2005 | Mobile phone | Yes | n.a. | Missing marital status |
| Senegal | 2010-11 | 2010-11 | 2005 | Mobile phone | Yes | n.a. | Missing marital status |
| Burundi | 2010 | 2016-17 | 2010 |  | No | Inconsistent regions |  |
| Congo (Brazzaville) | 2011-12 | 2011-12 | 2005 |  | No | Inconsistent regions |  |
| Madagascar | 2008-09 | 2008-09 | 2003-04 | Mobile phone | No | Inconsistent regions |  |
| Niger | 2012 | 2012 | 2006 |  | No | Inconsistent regions |  |
| Uganda | 2011 | 2016 | 2011 |  | No | Inconsistent regions |  |
| Zimbabwe | 2010-11 | 2015 | 2010-11 |  | No | Inconsistent regions |  |
| Guyana | 2009 | 2009 | none |  | No | Only 1 DHS round |  |
| Sao Tome and Principe | 2008-09 | 2008-09 | none |  | No | Only 1 DHS round |  |
| Tajikistan | 2012 | 2012 | none |  | No | Only 1 DHS round |  |
| Cote d’Ivoire | 2011-12 | 2011-12 | 1998-99 |  | No | Prior DHS is too old |  |
| Comoros | 2012 | 2012 | 1996 |  | No | Prior DHS is too old |  |
| Kyrgyz Republic | 2012 | 2012 | 1997 |  | No | Prior DHS is too old |  |
