# Supplementary material for: Access to environmental health assets across wealth strata: Evidence from 41 low- and middle-income countries
Source: PLoS One. 2018 Nov 16;13(11):e0207339. doi: 10.1371/journal.pone.0207339 (PMC6239312; doi:10.1371/journal.pone.0207339)
Supplement: S2 Table — (DOCX) [file pone.0207339.s002.docx]

**S2 Table.** Pooled multivariate regression estimates of the association between wealth and EHA access.

| VARIABLES | Piped water | Improved water | Improved sanitation | Improved fuel | Electricity | Bednet | Mobile phone |
| --- | --- | --- | --- | --- | --- | --- | --- |
|  |  |  |  |  |  |  |  |
| Wealth quintile | 0.036*** | 0.037*** | 0.067*** | 0.041*** | 0.086*** | 0.023*** | 0.10*** |
|  | (0.0042) | (0.0042) | (0.0082) | (0.0086) | (0.0085) | (0.0064) | (0.0049) |
| Urban | 0.23*** | 0.20*** | 0.15*** | 0.17*** | 0.36*** | 0.014 | 0.25*** |
|  | (0.029) | (0.032) | (0.020) | (0.033) | (0.040) | (0.019) | (0.023) |
| Female head of HH | 0.011* | 0.030*** | 0.013 | 0.0012 | 0.027*** | 0.017** | 0.0004 |
|  | (0.0059) | (0.0055) | (0.0082) | (0.0053) | (0.0053) | (0.0070) | (0.0056) |
| Age of head of HH | 0.0004 | 0.0006* | 0.0018*** | -0.0003 | -0.0000 | -0.0013*** | -0.0027*** |
|  | (0.0002) | (0.0003) | (0.0003) | (0.0002) | (0.0002) | (0.0004) | (0.0003) |
| Primary education – head of HH | 0.0058 | 0.030** | 0.012 | -0.020* | 0.044** | 0.031*** | 0.084*** |
|  | (0.012) | (0.014) | (0.014) | (0.011) | (0.017) | (0.0088) | (0.011) |
| Secondary education – head of HH | 0.031*** | 0.016 | 0.048*** | 0.043*** | 0.060*** | 0.027*** | 0.084*** |
|  | (0.0073) | (0.014) | (0.014) | (0.012) | (0.016) | (0.0091) | (0.013) |
| Tertiary education – head of HH | 0.054*** | -0.047*** | 0.087*** | 0.11*** | -0.0067 | 0.0051 | -0.024* |
|  | (0.019) | (0.013) | (0.023) | (0.023) | (0.020) | (0.010) | (0.013) |
| Previously married – head of HH | 0.0029 | 0.0075 | -0.0094** | -0.0027 | 0.0065* | -0.075*** | -0.029*** |
|  | (0.0037) | (0.0051) | (0.0040) | (0.0041) | (0.0036) | (0.0066) | (0.0048) |
| Never married – head of HH | 0.013 | -0.0003 | -0.045*** | -0.022 | 0.044*** | -0.18*** | 0.0088 |
|  | (0.0089) | (0.012) | (0.010) | (0.022) | (0.0088) | (0.014) | (0.0089) |
| Household size | 0.0001 | 0.0005 | 0.010*** | -0.007*** | 0.0003 | 0.0044** | 0.018*** |
|  | (0.0012) | (0.0010) | (0.0015) | (0.0013) | (0.0016) | (0.0018) | (0.0013) |
| Number of children | -0.007*** | -0.007*** | -0.014*** | 0.0029 | -0.012*** | 0.034*** | -0.023*** |
|  | (0.0018) | (0.0018) | (0.0024) | (0.0018) | (0.0026) | (0.0042) | (0.0030) |
| Constant | 0.036*** | 0.037*** | 0.067*** | 0.041*** | 0.086*** | 0.024*** | 0.10*** |
|  | (0.0042) | (0.0042) | (0.0082) | (0.0086) | (0.0085) | (0.0064) | (0.0049) |
|  |  |  |  |  |  |  |  |
| Observations | 497,477 | 497,490 | 497,556 | 478,629 | 497,419 | 348,481 | 497,421 |
| R-squared | 0.3947 | 0.1830 | 0.3897 | 0.4490 | 0.6013 | 0.1563 | 0.3876 |

Notes: Includes only the 41 countries having a DHS survey between 2008-2013 (for analogous results from the 29 countries with multiple rounds since 2000, refer to Table 3 in the main text). The regression specification in this table is a linear regression that includes year of survey and country fixed effects. Standard errors clustered at the country level are shown in parentheses. The wealth index used here is a country-specific index that was constructed using the first principle component obtained using PCA over all asset variables included in that country’s survey, excluding the outcome variables. Significance of the coefficients is indicated as follows: *** p<0.01; ** p<0.05; * p<0.1.
