# Supplementary material for: Access to environmental health assets across wealth strata: Evidence from 41 low- and middle-income countries
Source: PLoS One. 2018 Nov 16;13(11):e0207339. doi: 10.1371/journal.pone.0207339 (PMC6239312; doi:10.1371/journal.pone.0207339)
Supplement: S3 Table — (DOCX) [file pone.0207339.s003.docx]

**S3 Table.** Oaxaca-Blinder decomposition of the role of wealth disparities in differences of EHA ownership

| VARIABLES | Piped water | Improved water | Improved sanitation | Improved fuel | Electricity | Bed net | Mobile phone |
| --- | --- | --- | --- | --- | --- | --- | --- |
|  |  |  |  |  |  |  |  |
| Poor | 0.18*** | 0.64*** | 0.29*** | 0.090*** | 0.38*** | 0.50*** | 0.51*** |
|  | (0.044) | (0.035) | (0.042) | (0.023) | (0.059) | (0.039) | (0.039) |
| Non-poor | 0.30*** | 0.75*** | 0.52*** | 0.24*** | 0.65*** | 0.58*** | 0.81*** |
|  | (0.045) | (0.029) | (0.046) | (0.036) | (0.051) | (0.049) | (0.027) |
| Difference | -0.12*** | -0.11*** | -0.23*** | -0.15*** | -0.27*** | -0.084*** | -0.30*** |
|  | (0.016) | (0.015) | (0.019) | (0.020) | (0.027) | (0.020) | (0.020) |
| Endowments | -0.048*** | -0.021*** | -0.078*** | -0.062*** | -0.078*** | -0.006 | -0.056*** |
|  | (0.011) | (0.007) | (0.009) | (0.011) | (0.014) | (0.010) | (0.009) |
| Coefficients | -0.057** | -0.078*** | -0.15*** | -0.090*** | -0.16*** | -0.069*** | -0.20*** |
|  | (0.023) | (0.018) | (0.025) | (0.028) | (0.028) | (0.022) | (0.020) |
| Interaction | -0.018 | -0.014** | -0.003 | 0.004 | -0.025*** | -0.009 | -0.048*** |
|  | (0.013) | (0.006) | (0.014) | (0.012) | (0.0095) | (0.006) | (0.008) |
|  |  |  |  |  |  |  |  |
| Observations | 822,048 | 822,061 | 818,620 | 785,362 | 801,566 | 545,653 | 723,850 |

Notes: Includes both waves from the 29 countries with two rounds since 2000. Standard errors clustered at the country level are shown in parentheses, models include head of household characteristics and household demographic controls, as well as year of survey fixed effects. The wealth index used here is a country-specific index that was constructed using the first principle component obtained using PCA over all asset variables included in that country’s survey, only excluding the outcome variables. Poor are all households in the bottom two quintiles; non-poor are the others. Significance of the coefficients is indicated as follows: *** p<0.01; ** p<0.05; * p<0.1.
