# Supplementary material for: Access to environmental health assets across wealth strata: Evidence from 41 low- and middle-income countries
Source: PLoS One. 2018 Nov 16;13(11):e0207339. doi: 10.1371/journal.pone.0207339 (PMC6239312; doi:10.1371/journal.pone.0207339)
Supplement: S4 Table — (DOCX) [file pone.0207339.s004.docx]

**S4 Table.** Pooled multivariate regression estimates of the association between wealth and EHA access, allowing for country-specific differences.

| VARIABLES | Piped water | Improved water | Improved sanitation | Improved fuel | Electricity | Bed net | Mobile phone |
| --- | --- | --- | --- | --- | --- | --- | --- |
|  |  |  |  |  |  |  |  |
| Wealth quintile | 0.039*** | 0.036*** | 0.066*** | 0.040*** | 0.090*** | 0.025*** | 0.10*** |
|  | (0.0042) | (0.0043) | (0.0060) | (0.0070) | (0.0088) | (0.0058) | (0.0056) |
| Urban | 0.23*** | 0.20*** | 0.15*** | 0.17*** | 0.36*** | 0.014 | 0.25*** |
|  | (0.030) | (0.0321) | (0.0206) | (0.0334) | (0.0413) | (0.020) | (0.023) |
| Constant | 0.16 | 0.71*** | 0.069 | -0.087** | 0.13 | 0.50*** | -0.067 |
|  | (0.22) | (0.054) | (0.26) | (0.044) | (0.27) | (0.020) | (0.048) |
| Cross-country variance – wealth | 0.0008*** | 0.0008** | 0.0015*** | 0.0019*** | 0.0028*** | 0.0009** | 0.0012*** |
|  | (0.0002) | (0.0002) | (0.0002) | (0.0005) | (0.0006) | 0.0004 | (0.0003) |
| Cross-country variance – constant | 0.035*** | 0.030*** | 0.061*** | 0.031** | 0.081*** | 0.036*** | 0.047*** |
|  | (0.013) | (0.0063) | (0.017) | (0.015) | (0.020) | (0.0095) | (0.0090) |
|  |  |  |  |  |  |  |  |
| Observations | 497,477 | 497,490 | 497,556 | 478,629 | 497,419 | 348,481 | 497,421 |

Notes: Includes only the 41 countries having a DHS survey between 2008-2013 (for analogous results from the 29 countries with multiple rounds since 2000, refer to Table 5 in the main text). Standard errors clustered at the country level are shown in parentheses, models include head of household characteristics and household demographic controls, as well as year of survey fixed effects and random intercept and wealth slopes. The wealth index used here is a country-specific index that was constructed using the first principle component obtained using PCA over all asset variables included in that country’s survey, only excluding the outcome variables. Significance of the coefficients is indicated as follows: *** p<0.01; ** p<0.05; * p<0.1.
