# Supplementary material for: Access to environmental health assets across wealth strata: Evidence from 41 low- and middle-income countries
Source: PLoS One. 2018 Nov 16;13(11):e0207339. doi: 10.1371/journal.pone.0207339 (PMC6239312; doi:10.1371/journal.pone.0207339)
Supplement: S5 Table — (DOCX) [file pone.0207339.s005.docx]

**S5 Table.** Pooled multivariate regression estimates of the association between wealth and EHA access, from regional-level panel analysis

| VARIABLES | Piped water | Improved water | Improved sanitation | Improved fuel | Electricity | Bed net | Mobile phone |
| --- | --- | --- | --- | --- | --- | --- | --- |
| Random effects specification |  |  |  |  |  |  |  |
| Wealth quintile | 0.033 | 0.078*** | 0.051*** | 0.070*** | 0.088*** | -0.0034 | 0.098*** |
|  | (0.023) | (0.022) | (0.019) | (0.019) | (0.016) | (0.032) | (0.015) |
| Urban | 0.12** | 0.056 | 0.082 | 0.29*** | 0.37*** | -0.028 | 0.20*** |
|  | (0.063) | (0.053) | (0.071) | (0.083) | (0.10) | (0.11) | (0.043) |
| Constant | -0.029 | 0.34* | -0.12 | 0.12 | -0.015 | 0.51** | -0.53*** |
|  | (0.17) | (0.20) | (0.16) | (0.17) | (0.22) | (0.26) | (0.18) |
| Cross-country variance – wealth | 0.0096*** | 0.0037 | 0.0052*** | 0.0071 | 0.0053** | 0.0010 | 0.0011 |
|  | (0.0035) | (0.0036) | (0.0022) | (0.0054) | (0.0024) | (0.0012) | (0.0011) |
| Cross-country variance – constant | 0.11*** | 0.047 | 0.064*** | 0.050 | 0.11** | 0.019*** | 0.023 |
|  | (0.046) | (0.038) | (0.026) | (0.058) | (0.045) | (0.0090) | (0.019) |
|  |  |  |  |  |  |  |  |
| Observations | 577 | 577 | 587 | 579 | 568 | 382 | 495 |
| Fixed effects specification |  |  |  |  |  |  |  |
| Wealth quintile | 0.0047 | 0.046 | 0.029 | 0.038 | 0.045 | 0.10 | 0.094 |
|  | (0.033) | (0.048) | (0.031) | (0.037) | (0.030) | (0.10) | (0.1) |
| Urban | 0.10 | 0.22 | 0.23* | 0.22* | 0.23** | 0.46* | 0.061 |
|  | (0.11) | (0.20) | (0.1161) | (0.11) | (0.11) | (0.26) | (0.66) |
| Constant | 0.043 | 0.45 | -0.34 | -0.55 | -0.73 | -1.06 | -0.31 |
|  | (0.30) | (0.66) | (0.40) | (0.59) | (0.45) | (1.31) | (1.56) |
|  |  |  |  |  |  |  |  |
| Observations | 577 | 577 | 587 | 579 | 568 | 382 | 495 |

Notes: Includes the 29 countries having multiple DHS surveys after the year 2000. Standard errors clustered at the country level are shown in parentheses, models include all controls from previous analyses, aggregated to the region level, as well as year of survey fixed effects. The random effects specification allows for country-specific slopes and intercepts; the fixed effects specification uses region fixed effects. The wealth index used here is a country-specific index that was constructed using the first principle component obtained using PCA over all asset variables included in that country’s survey, only excluding the outcome variables. Significance of the coefficients is indicated as follows: *** p<0.01; ** p<0.05; * p<0.1.
